# A Case of Extrusion of a Solid Silicone Tire Migrating through the Superior Rectus Muscle with *Aeromonas hydrophila* Infection following a Scleral Buckling Procedure

**DOI:** 10.1155/2012/167164

**Published:** 2012-11-20

**Authors:** Shinji Makino, Yukihiro Sato

**Affiliations:** Department of Ophthalmology, Jichi Medical University, 3311-1 Yakushiji, Tochigi, Shimotsuke 329-0498, Japan

## Abstract

To our knowledge, there are no reports of *Aeromonas hydrophila* infection after a scleral buckling procedure. Also, migration of a silicone explant element through the rectus muscles is extremely rare. Herein, we describe a case experiencing extrusion of a solid silicone tire migrating through the superior rectus muscle with *Aeromonas hydrophila* infection following a scleral buckling procedure. A 42-year-old man was referred to our hospital complaining of ocular pain and purulent discharge in his left eye which has persisted for several months. He had a history of bilateral rhegmatogenous retinal detachment which had been treated with scleral buckling. The left eye showed extrusion of the solid silicone buckle which had migrated through the superior rectus muscle and an infection in the upper quadrant of the sclera. The buckle was removed, and the patient was treated with antibiotics. After the removal of the buckle, the symptoms showed rapid amelioration and there was no recurrence of retinal detachment. *Aeromonas hydrophila* was isolated from the discharge and the removed explant. He used well water in daily life. In this case, the *Aeromonas hydrophila* infection of the extruded buckle might have originated from contaminated well water.

## 1. Introduction

The *Aeromonas* genus consists of Gram-negative bacilli, found throughout the environment, particularly in water and soil. Only the three phenospecies, *Aeromonas hydrophila*, *Aeromonas sorbia*, and *Aeromonas caviae*, are of clinical significance in humans [1]. *Aeromonas hydrophila *infections usually occur under specific circumstances, such as in immunocompromised patients, especially those with malignancy or liver diseases. *Aeromonas hydrophila* has been reported to cause conjunctivitis [2], corneal ulcer [3, 4], contact lens-related keratitis [5], orbital cellulitis [6], and endophthalmitis [7–12]. To our knowledge, there are no reports of *Aeromonas hydrophila* infection after a scleral buckling procedure.

Extrusion and infection of the buckle are infrequent complications of scleral buckling procedures [13–18]. However, there are a few reported cases describing migration of a silicone explant element through the rectus muscles [19–21].

We present a case with extrusion of a solid silicone tire migrating through the superior rectus muscle with *Aeromonas hydrophila* infection following a scleral buckling procedure.

## 2. Case Report

**Figure 1:**
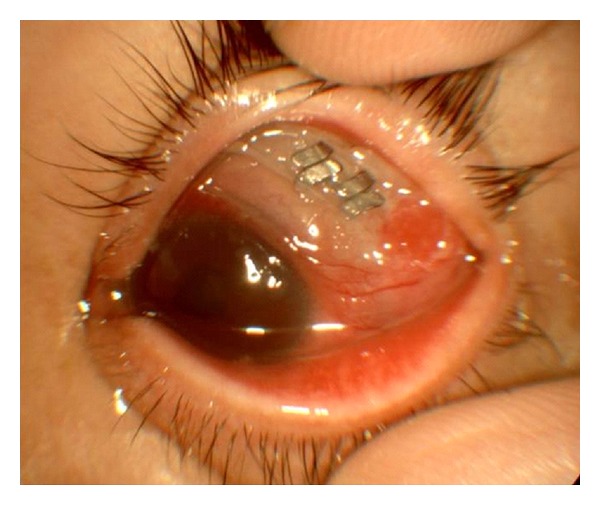
Photograph of the left eye at the time of presentation. An extrusion of the buckle which had migrated through the superior rectus muscle and infected sclera was observed through the conjunctival fistula.

**Figure 2:**
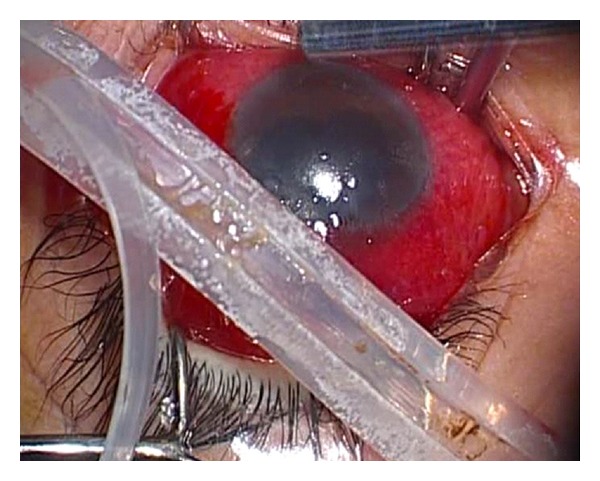
Photograph of the removed buckle. Mucopurulent pus is seen around the buckle.

A 42-year-old man presented with ocular pain, hyperemic swollen conjunctiva, and purulent discharge in his left eye which lasted for several months prior to his first visit to our facility. His ocular history was significant for bilateral rhegmatogenous retinal detachment 16 years prior to the current presentation. The right eye had been successfully treated with a scleral buckling procedure and vitrectomy 9 years earlier. The left eye underwent a scleral buckling procedure with placement of a 270°-solid silicone explant (MIRA number 277) with a 360°-encircling band (MIRA number 240). Upon examination on presentation, visual acuity was 20/20 in the right eye and 20/200 in the left eye. The anterior segment of the right eye showed postoperative aphakia, and ophthalmoscopy revealed an attached retina with indentation of the scleral buckle at 360°. The left eye demonstrated hyperemic and chemotic conjunctiva and extensive yellowish discharge. Conjunctival fistula was present in the upper quadrants. An extrusion of the buckle had migrated through the superior rectus muscle and scleral infection was observed through the conjunctival fistula (Figure 1). An encircling band and a solid explant were removed (Figure 2), and the patient was treated with topical and systemic broad spectrum antibiotics. After the removal of the buckle, the symptoms improved rapidly and there was no recurrence of retinal detachment. His visual acuity improved to 60/200. *Aeromonas hydrophila *was isolated from the discharge and the removed explant. He used well water in daily life. *Aeromonas hydrophila* was not, however, isolated from the well water. 

## 3. Discussion

To our knowledge, this is the first case report describing late infection caused by *Aeromonas hydrophila* following a scleral buckling procedure.

Among the infrequent complications associated with scleral buckling procedures are inflammation, infection, extrusion, and intrusion [13–18]. Postoperative extrusion or infection with exposure of the scleral buckling material is reportedly more common with the use of silicone sponge explants (2.7%–18.0%) than with that of hard silicone explants (0.2%–1.4%) [14]. 

These complications also are more common in patients with explants than in those with implants. However, a few cases with extrusion and anterior displacement of a buckle migrating through the rectus muscles have been reported [19–21]. In our case, it was thought that the buckle had migrated though the superior rectus muscle. We speculated that anterior displacement of the buckle had eroded the superior rectus muscle first, followed by readhesion of the superior rectus muscle to the sclera and extrusion of the buckle. 

Folk et al. [13] reported the incidence of scleral abscess after buckling procedures to be 0.58%. Of the bacteria identified in scleral buckling infections, coagulase-negative *Staphylococcus* species are the most common, and in acute-onset scleral explant infection, which is relatively common, *Staphylococcus aureus*, *Staphylococcus epidermidis*,* Proteus mirabilis*, and *Pseudomonas aeruginosa* are usually identified [13–18]. However, according to our literature search, there have been no reports of *Aeromonas hydrophila* infection after a scleral buckling procedure. In our present case, *Aeromonas hydrophila* infecting the extruded buckle might have been derived from well water. *Aeromonas* species were reportedly isolated from 43 of 157 (27.4%) well water samples, and 36 of these 43 (83.7%) were *Aeromonas hydrophila* [22]. Although these organisms are found throughout the year, isolation ratios were higher in the summer (44.7%) than in the winter (17.2%) months. 

Thus, we speculated that undetected *Aeromonas hydrophila* from the well water used by this patient had caused the infection prior to testing, at a time when bacterial levels in the water would have been higher. *Aeromonas hydrophila* is highly pathogenic especially to the eye, triggering a robust inflammatory response with a rapid clinical course causing extensive necrosis with suppurative inflammation, and has a poor visual prognosis.

In conclusion, the possibility of an *Aeromonas* infection should be considered in cases with buckle infections, especially in the presence of environmental factors such well-water use in daily life.
